# Disulfide bond reduction of antibodies during clarification: A bench‐scale assay and a mitigation tool

**DOI:** 10.1002/btpr.88507

**Published:** 2026-04-03

**Authors:** Hendri Tjandra, Deepa Surendar, Isu Yoon, Florence Rusly, Yan Su, Vu Thai, Yi Liu, Ashley Hesslein

**Affiliations:** ^1^ Biologics Pilot Lab PDM, Gilead Sciences Foster City California USA; ^2^ Biologics Development Isolation & Purification, Bayer Healthcare LLC Berkeley California USA; ^3^ Manufacturing Science and Technology – Purification Lonza Vacaville California USA; ^4^ Pre‐Pivotal Purification Development PDM, Gilead Sciences Foster City California USA; ^5^ Process Development Ultragenyx Pharmaceutical Inc. South San Francisco California USA

**Keywords:** antibody integrity, biomanufacturing scale‐up, chromatographic primary recovery, depth filtration differential pressure, disulfide bond reduction (DSBR), harvest clarification, scale‐down model assay

## Abstract

We investigated side‐by‐side clarification with a two‐stage depth filter system against a single‐stage charge chromatographic clarifier on a number of Chinese hamster ovary (CHO) cell cultures. We found that the one‐stage fibrous anion exchange (AEX) clarification met or exceeded the performance of the conventional two‐stage depth filtration systems in terms of product recovery, removal of process‐related impurities, and throughput. The one‐stage anion clarification approach had a smaller footprint, a shorter set‐up time, an easier setup, consumed less buffer flush volume, and operated at a lower differential pressure relative to those of conventional systems. In a comparability study at 200 L scale, antibodies in cell culture fluid (CCF) clarified by depth filtration system exhibited product quality degradation due to disulfide bond reduction (DSBR), whereas antibodies clarified by AEX fiber method were not. We surmised that the lower operating differential pressure and electrostatic adsorption mechanism of the anion clarification system minimized the potential of product to undergo DSBR. We developed a bench‐scale process model that allowed us to assay DSBR and predict the process results. We then utilized it to demonstrate that the antibody was reduced when clarified with a two‐stage 3 M™ Zeta Plus™ Depth Filter system but not with the anionic clarification system. This bench‐scale DSBR assay allowed us to rank the relative disulfide bond reduction potentials of various monoclonal antibodies (mAbs) and demonstrate the reduction‐mitigating impacts of the fibrous clarification approach on a reduction‐prone antibody culture at 2000 L processing scale.

## 1 INTRODUCTION

Cell culture fluid harvest unit operation is the first step in the purification of recombinant proteins following their expression in Chinese Hamster Ovary (CHO) cells. Over the years, several different strategies and approaches have been developed to accomplish this task. Initially, the primary method of harvesting was clarification of cell culture fluid (CCF) using a combination of depth filtration and centrifugation.^1^, ^2^ Depth filtration is based primarily on separation by size through a paper‐like wet‐laid medium, and centrifugation is based on separation by density. A few commercial improvements were developed by industrial filtration manufacturers that offer various advantages of encapsulated filtration media and continuous centrifugation,^3^ but both filtration and centrifugation are limited to separation of large (> 0.5 μm) insoluble particles such as cells, cell debris, and bio‐molecular aggregates.^4^, ^5^ The technology solution incorporating these methods works well for manufacturing processes with low cell densities and stable products such as classical IgG_1_, IgG_2_, IgG_4_ mAbs, small enzymes and cytokines.

As the cell culture densities increased to boost titers and the biopharma product candidate pipelines filled with a variety of molecules, including designer mAbs and antibody derivatives, the expectations of the harvest and purification process strategy were more stringent. Classic clarification and removal of insoluble particles are no longer sufficient, as the removal of key soluble components takes a pivotal role during harvest to increase the stability of clarified cell culture fluid (CCCF), increase consistency of sterile filter life, and improve performance of downstream capture and polishing chromatography.^5^, ^6^, ^7^ Clarification technology and strategy evolved together with the goals and expectations of the industry. Harvest strategy started to include chromatographic approaches to deal with higher cell mass, which results in more cell debris and increased soluble impurities in the CCF. These systems utilize fibrous AEX technology to capture negatively charged contaminants during harvest rather than clarify using size or density differences.^4^, ^5^, ^7^ The result is that the amount of insoluble and key soluble impurities in the CCCF decreased by orders of magnitude.

One other important aspect of harvest and clarification development strategy is exploring approaches and technologies to reduce cell shear and product degradation. One approach to control degradation during the harvest is by reducing the exposure of product to impurities that result from cell shear and lysis during the harvest. The increasing diversity of designer antibodies and antibody derivatives in the biopharma pipeline is expected to result in a broader range of product sensitivities. Since conventional depth filtration relies on trapping cells in the pores of the wet‐laid media and continuous centrifugation relies on the introduction of cells into fast moving streams and settling field inside disk stack centrifuges, the impacts of cell shear and cell lysis should be considered when harvesting. Minimizing cell shear requires special consideration when designing and executing process strategy.^8^, ^9^, ^10^, ^11^ Newer anion exchange chromatography methods utilize open fiber matrices that trap cells by electrostatic charge and operate at low pressure (< 5 psi) to minimize cell shear altogether. Here we explore and compare the effect of using different clarification approaches on cell shear and product quality.

We show that a single‐stage AEX fiber clarification system achieves product recovery and impurity removal comparable to, or better than, conventional two‐stage depth filtration, while offering operational advantages. Through comparability studies and a newly developed bench‐scale assay, we demonstrate that the AEX approach effectively mitigates disulfide bond reduction in antibody cultures—a challenge frequently observed with depth filtration when scaling up clarification during manufacturing. Apart from the observed differences in disulfide bond reduction, product quality attributes were otherwise comparable between depth filtration and AEX fiber clarification. Our findings highlight the importance of controlling clarification end‐point differential pressure to prevent DSBR while maintaining manufacturable throughput.

## 2 RESULTS AND DISCUSSIONS

### 2.1 Evaluation of harvest strategy using two stages of depth filtration versus single stage AEX fiber matrix

**FIGURE 1:**
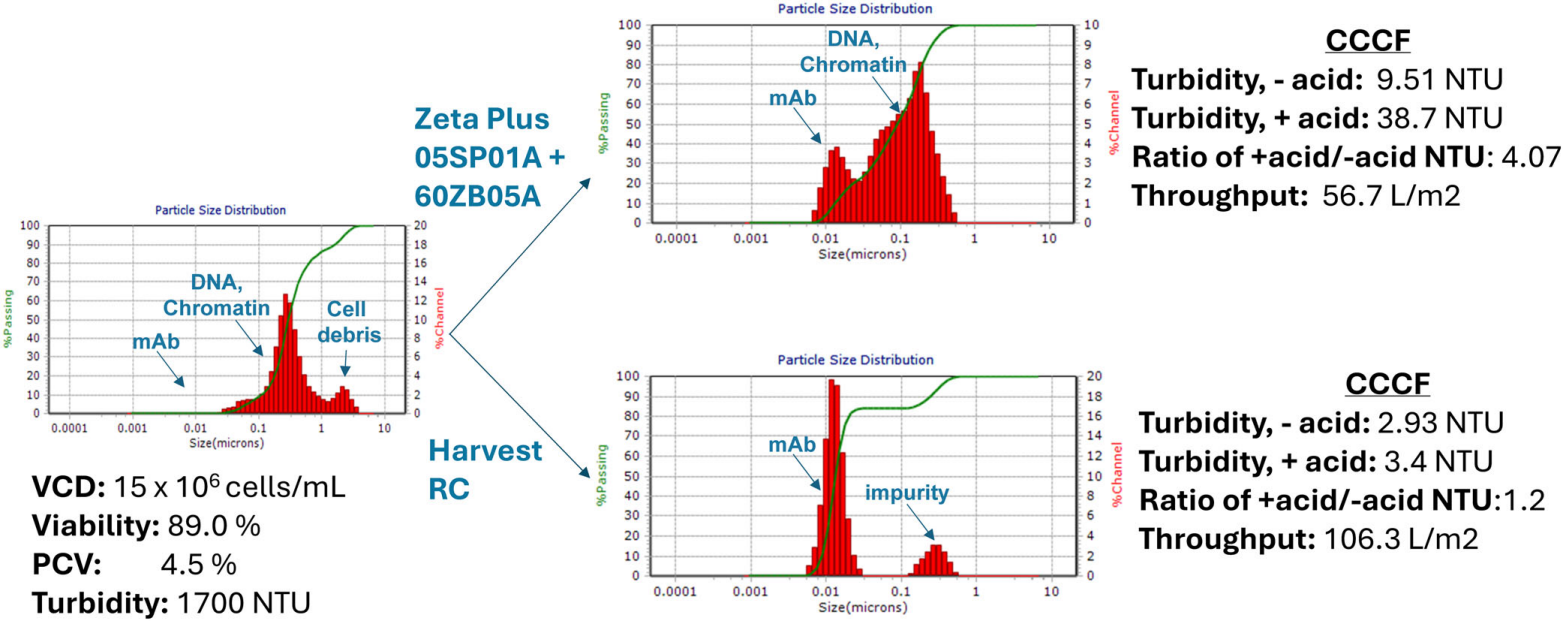
Particle size distribution and turbidity readouts following clarification with 3 M™ Zeta Plus™ Depth Filter (top) or 3 M™ Harvest RC Chromatographic Clarifier (bottom).

An AEX fiber clarification system (3 M™ Harvest RC Chromatographic Clarifier) was evaluated as a clarification tool to harvest fluid from eight cell cultures of different cell densities expressing IgG_1_, IgG_2_ and IgG_4_ isotypes. Previously, a number of these cell cultures have been clarified with legacy depth filters such as the two‐stage 3 M™ Zeta Plus™ Depth Filter 05SP01A and 60ZB05A combination with or without the 3 M™ Emphaze™ AEX Hybrid Purifier fiber clarifier. As such, we carried out side‐by‐side performance evaluations of legacy wet‐laid depth filters with the new chromatographic solution. In one such evaluation, we compared the performance of the approaches on a cell culture expressing antibody “mAb D”. As shown in Figure 1, the depth filter combination of 3 M™ Zeta Plus™ Depth Filter 05SP01A and 60ZB05A could enrich the antibody pool centered around 0.01 μm following clarification. However, a DLS (Dynamic Light Scattering) peak corresponding to particle size range between 0.08 to 0.8 µm remained a significant fraction of clarified cell culture fluid (CCCF). This filtrate sub‐population is likely chromatin hetero‐aggregates previously described by Gagnon and co‐workers.^6^ In contrast, the cell culture clarified with the anionic fiber clarification system showed a significant enrichment of the DLS peak centered around 0.01 μm that corresponded to the size of the antibody product. There was a relatively miniscule peak in the 0.1–0.5 μm. The volumetric throughput of 3 M™ Harvest RC was 106 L/m^2^ while that of 3 M™ Zeta Plus™ Depth Filter 05SP01A was 85 L/m^2^ and 60ZB05A at 170 L/m^2^ at their terminal end points. At a 2‐to‐1 filtration area ratio of 3 M™ Zeta Plus™ Depth Filter 05SP01 and 60ZB05 combination, the overall throughput of the depth filtration was thus 56.7 L/m^2^. As such, the single‐stage AEX fiber clarification system uses a smaller total frontal surface area and occupies a smaller footprint when scaling up to 2000 L cell culture scale. Additionally, the turbidity of the CCCF, which was indicative of CHO Host Cell DNA (HCD) and Host Cell Proteins (HCP), was 2.9 NTU while that of the 3 M™ Zeta Plus™ Depth Filter 05SP01A + 60ZB05A combination was 9.5 NTU. Upon acidification, the respective CCCF had a ratio of 1.2 for the anionic filtrate and 4.07 for the depth filtration filtrate.^4^ This comparison showed that the single‐stage charge filtration system required smaller filter area and removed more process‐related impurities. This translates into a cleaner feed stream for the subsequent affinity capture step and possibly further downstream step(s) such as neutralization of Protein A eluate following low pH viral inactivation.

### 2.2 Comparable product attributes of antibodies clarified by depth filters or charge filters

**FIGURE 2:**
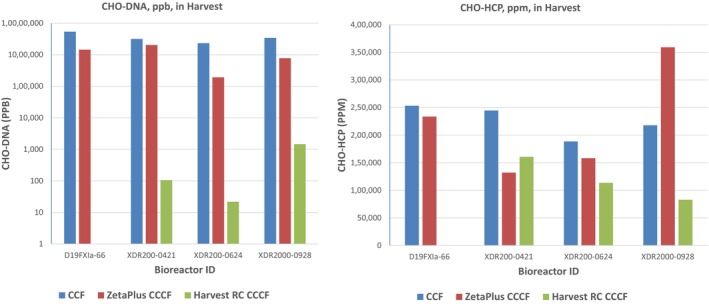
Impurity removal by either filtration system. The three batches of CCCF generated by either 3 M™ Zeta Plus™ Depth Filters (red bars) or 3 M™ Harvest RC Chromatographic Clarifier (green bars) were assayed for Host Cell DNA (left panel) or Host Cell Proteins (right panel). The control batch D19 was clarified by only 3 M™ Zeta Plus™ Depth Filters.

Next, the cell culture fluid clarified by either depth filter or AEX fiber matrix was purified on a Protein A Mab Select SuRe LX (Cytiva, USA) column for further analysis. As shown on Figure 2a, the DNA impurities in the starting CCCF from 200 to 2000 L runs were higher in those clarified with depth filter. This was not surprising as the quaternary ammonium (Q) functionality on the fiber clarification matrix was very effective at binding highly negatively charged DNA at cell culture harvest conditions. In contrast, the removal of HCP from this cell culture by either filtration system was comparable.

**FIGURE 3:**
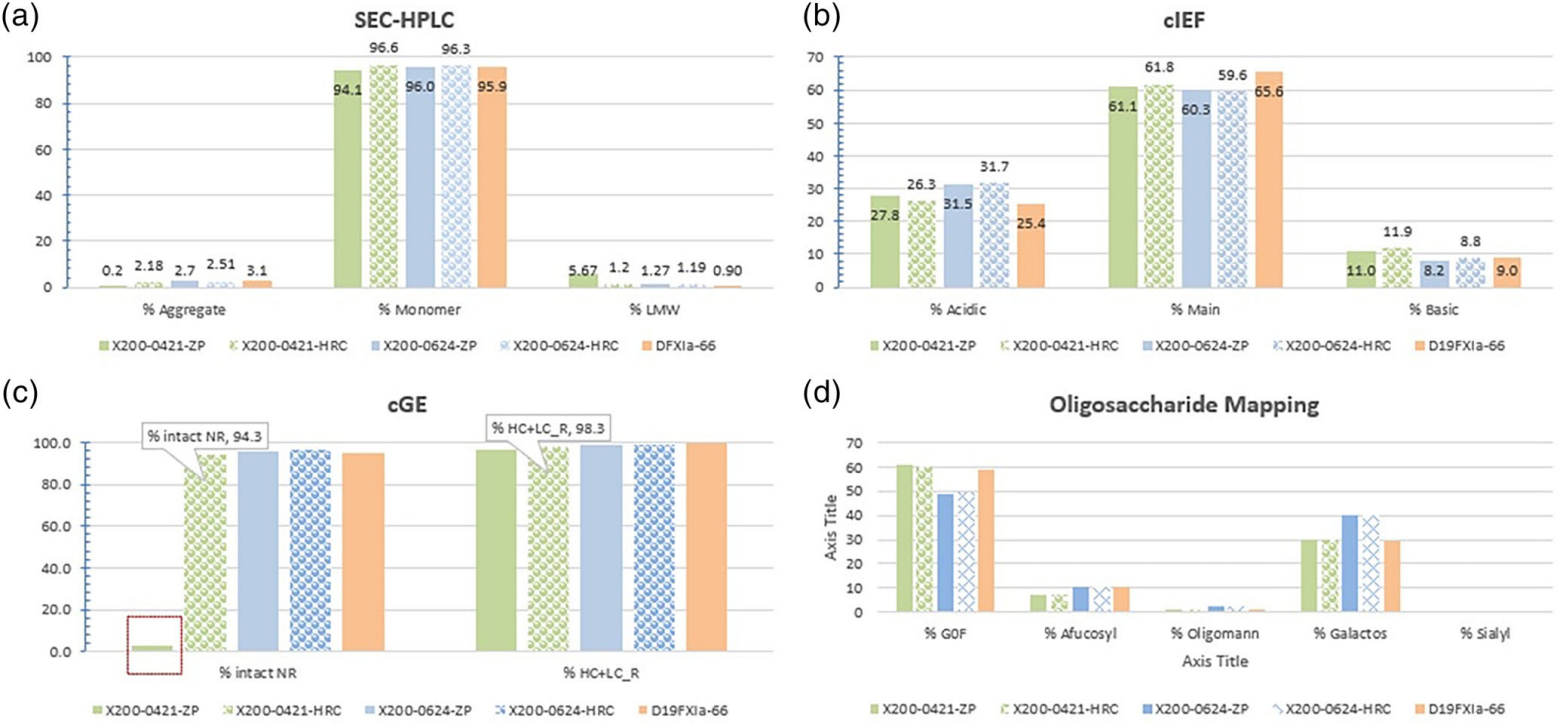
(a–d) Two batches of CCCF generated either 3 M™ Zeta Plus™ Depth Filter (solid bars) or by 3 M™ Harvest RC Chromatographic Clarifier (checkered bars) as well a control reference batch, D19, were affinity purified on Protein A column. Protein A eluate was then assayed for monomers (by SEC‐HPLC), charged variants (by iCIEF), purity (by cGE) and oligosaccharide (by Oligo Map).

**FIGURE 4:**
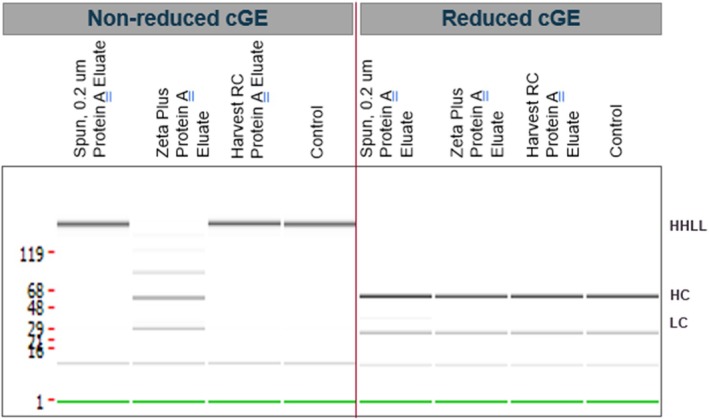
Harvest cell culture fluid from a 200 L bioreactor was clarified by centrifugation, 3 M™ Zeta Plus™ Depth Filter, or 3 M™ Harvest RC Chromatographic Clarifier. The CCCF samples were then affinity‐purified and analyzed on non‐reducing (left panel) or reducing cGE (right panel).

When the CCCF, clarified by either one of the above approaches, was purified on an affinity Protein A column and analyzed for product attributes, both appeared to be comparable in the monomer content (by SEC‐HPLC), charged variants (by iCIEF), oligosaccharide (by Oligo Map) and purity (by cGE). However, there were only 2.8% intact antibodies in the CCCF harvested by the depth filter compared to 94.3% intact antibodies in the CCCF harvested by the AEX fiber (Figure 3c). The total amount of purified mAb heavy chain and light chain in either CCCF was comparable, suggesting that 2.8% intact observed in depth filter‐clarified cell culture fluid was likely due to DSBR of antibodies reported to occur in large‐scale clarification and during the storage of antibodies prior to affinity Protein A capture.^10^, ^12^ Further analysis of CCCF spun down by centrifugation and affinity Protein A‐purified showed that the disulfide bonds of the antibody were intact prior to the depth filtration step (Figure 4).

### 2.3 Bench‐scale reduction model

Until recently, there had not been a simple bench‐scale model assay available as a tool to evaluate and estimate the disulfide bond reduction (DSBR) potential of a CHO culture. Building on the comparability results above, we observed that depth filtration exposed certain antibody products to DSBR whereas AEX fiber clarification did not. Based on this observation, we developed a bench‐scale process model to predict DSBR risk early in process development. This model leverages scale dependent clarification effects observed during manufacturing. Its goal is to reproduce, at bench scale, the mechanistic conditions present during large‐scale clarification and subsequent process step hold, enabling identification of reduction‐prone antibodies and mitigation of quality risks prior to scale‐up.

#### 2.3.1 Mechanistic rationale

DSBR is driven by thioredoxin−/glutathione‐mediated pathways upon shear‐ or pressure‐induced cell lysis, with NADPH sustaining the reductive environment.^12^, ^13^, ^14^ At the manufacturing scale clarification, a higher differential pressure (ΔP) across the stacked filters, cake formation, and relatively longer hold time promote release of reductases and localized oxygen depletion. These conditions collectively favor thiol‐disulfide exchange.^10^, ^12^, ^13^

#### 2.3.2 Model design

**FIGURE 5:**
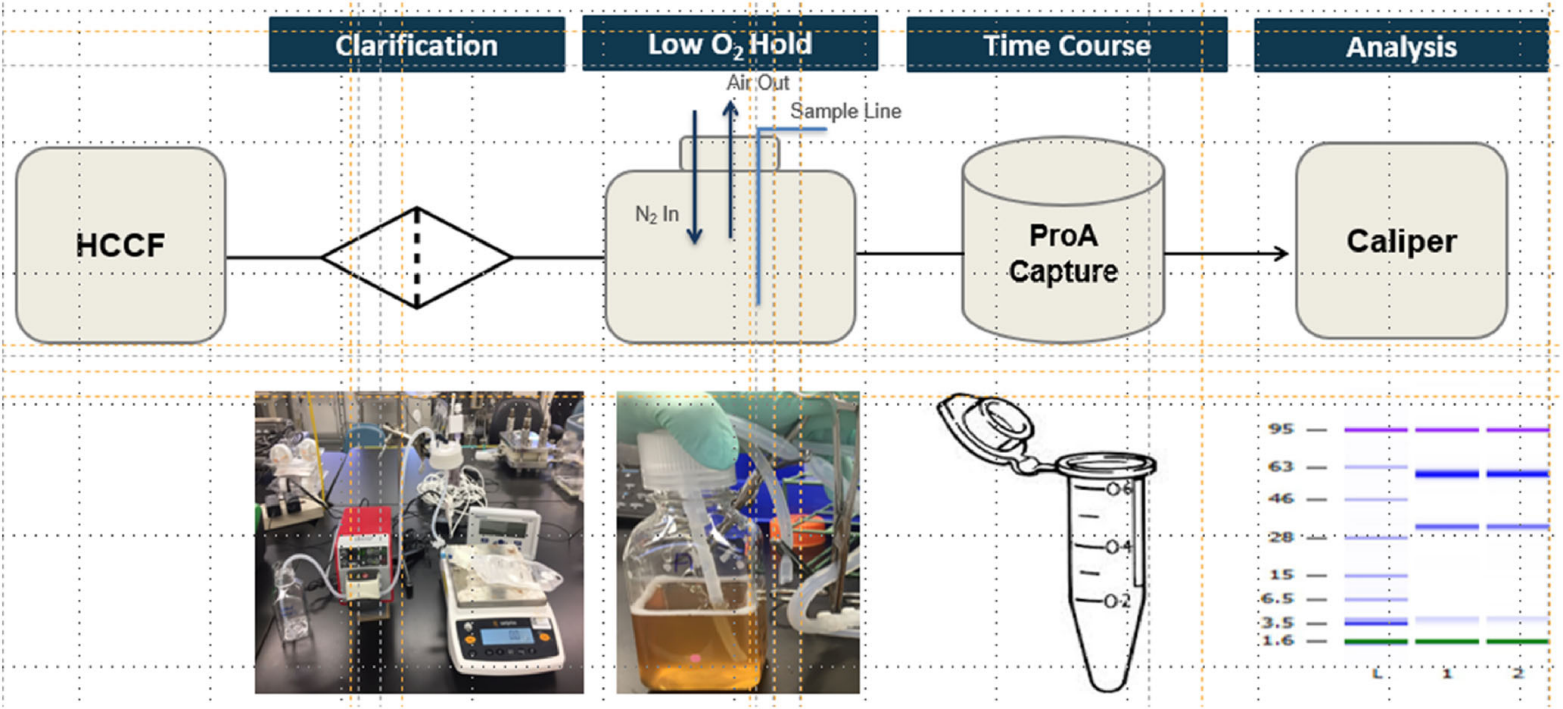
Schematic of a bench‐scale process model and assay to predict disulfide bond reduction.

Trexler‐Schmidt et al. modeled the potential or likelihood of DSBR in cell culture by mechanically homogenizing intact cells and adding the homogenate to reconstitute a DSBR event in an intact cell culture.^13^ Similarly, O'Mara et al. found that DSBR could be attenuated by carefully selecting a certain combination of 3 M™ ZetaPlus™ depth filters.^10^ In our studies to predict the likelihood of DSBR occurring at manufacturing scale, we developed a bench‐scale process model that reliably mimics the DSBR potential using scale‐down depth filters with cell cultures from 2 to 2000 L bioreactors. To mirror the large‐scale depth filtration conditions, we clarified CCF on a 22‐cm^2^ Cytiva PDK5 (Cytiva, USA) depth filter at 3 different fluxes (low, middle, and high) chosen to replicate manufacturing‐scale ΔP profiles. The PDK5 filter was chosen as the model assay filter as we initially observed antibody “mAb B” undergoing DSBR when depth filtered with PDK5 at 2000 L scale. Additionally, PDK5 filter is a two‐stage‐in‐one capsule (with nominal pore‐size ranges of ~20–10 μm and ~ 3.5–1.5 μm). It is a simpler setup relative to assembling two separate depth filter capsules connected in series (e.g., 10SP02 with ~7 to ~1 μm and 60ZB05 with 3 to 0.3 μm pore size). The PDK5 filtrates were held in low‐oxygen environment (e.g., sealed containers) and purged with N_2_ to remove oxygen to emulate low dissolved oxygen (DO) post‐clarification hold conditions. This setup preserves a reductive environment and allows DSBR to manifest when the cell culture is predisposed. Time‐point samples were collected to mimic manufacturing hold times, Protein A affinity purified, and the intactness of the antibody was assessed and quantified (Figure 5).

#### 2.3.3 Interpretation across filter trains: differential pressure and pore‐size effects

**FIGURE 6:**
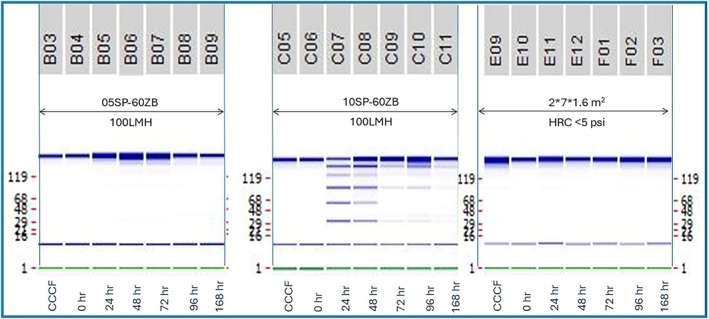
MAb “D” culture was clarified with a combination of 3 M™ Zeta Plus™ Depth Filter 05SP01A + 60ZB05A (left panel), 10SP02A + 60ZB05A (middle panel) at 100 LMH flux to NMT 15 psi, or by 3 M Harvest RC (right panel) to 3 psi then blown down to a terminal differential pressure, ΔP, of 5 psi. The CCCFs were kept in an oxygen‐depleted environment, time‐point samples collected at 24, 48, 72, 96, 168 h before further purification on Protein A affinity resin and separated on a cGE.

The bench‐scale assay is built around reproducing the conditions under which DSBR is observed at scale—specifically elevated differential pressure with cake compaction that can increase cell lysis, followed by a low‐oxygen post‐clarification hold. Because depth filters differ in nominal pore range and architecture, they do not generate the same differential pressure behavior at a fixed flux for a given culture and throughput endpoint. More restrictive pore ranges are more likely to produce elevated differential pressure and increased cell lysis, which are conditions associated with DSBR. Consistent with prior reports and with our comparisons in Figure 6, restrictive pore‐size depth filters are more likely to generate higher differential pressure and DSBR, while larger pore‐size filters may not. Accordingly, PDK5 is used here as a reproducible bench‐scale “stress generator” to achieve manufacturing‐relevant ΔP/cake formation. As such, the model's prediction should be interpreted as relative DSBR risk under clarification conditions that drive elevated differential pressure and cell lysis.

**FIGURE 7:**
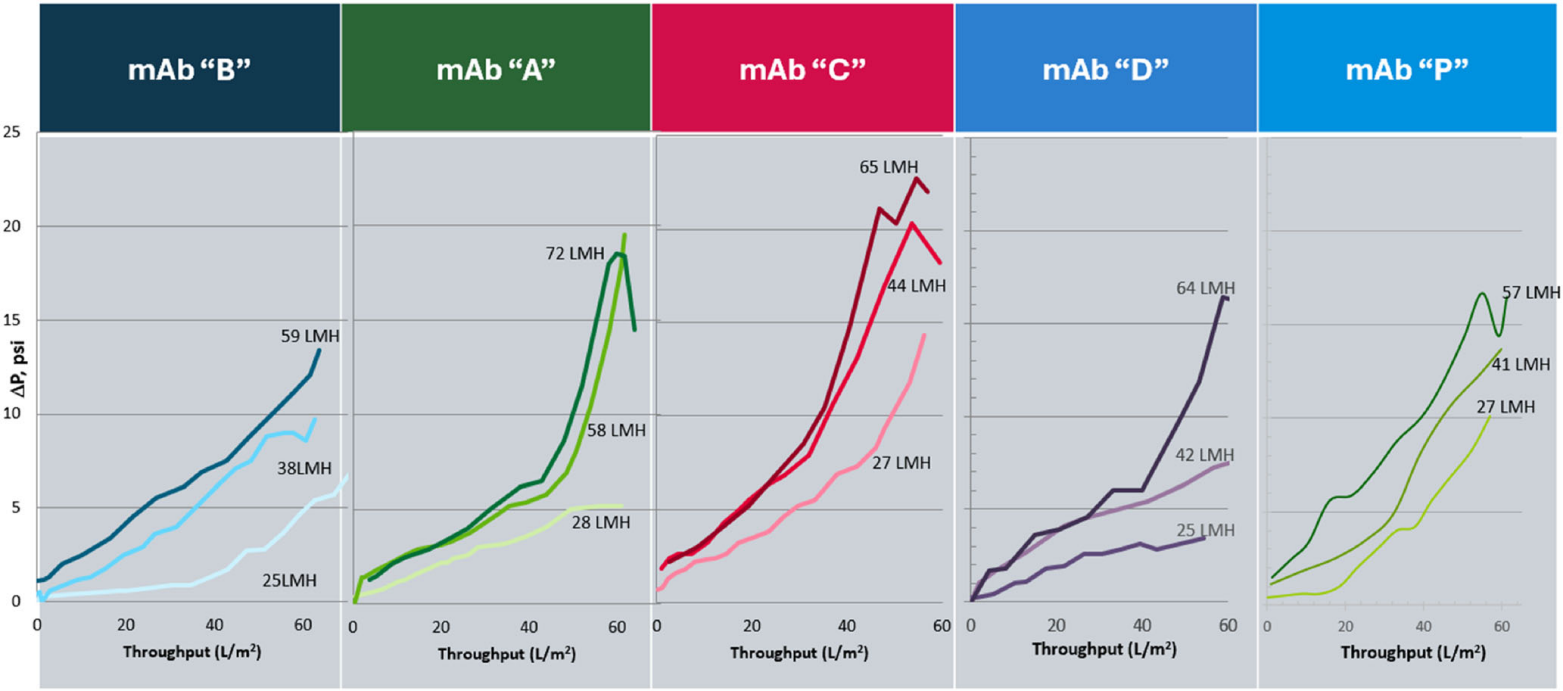
Differential pressure curves generated by various CHO cultures when clarified at three fluxes.

Shown in Figure 7 are the differential pressure curves generated by individual cell culture when clarified at three different filtration fluxes on a Cytiva PDK5 filter. Filtration fluxes of 25, 50, and 70 LMH were targeted for each of these cell cultures to mimic the differential pressure that is generated at 200–2000 L scale when they are typically clarified at 30–50 LMH.

#### 2.3.4 Assay readout

**FIGURE 8:**
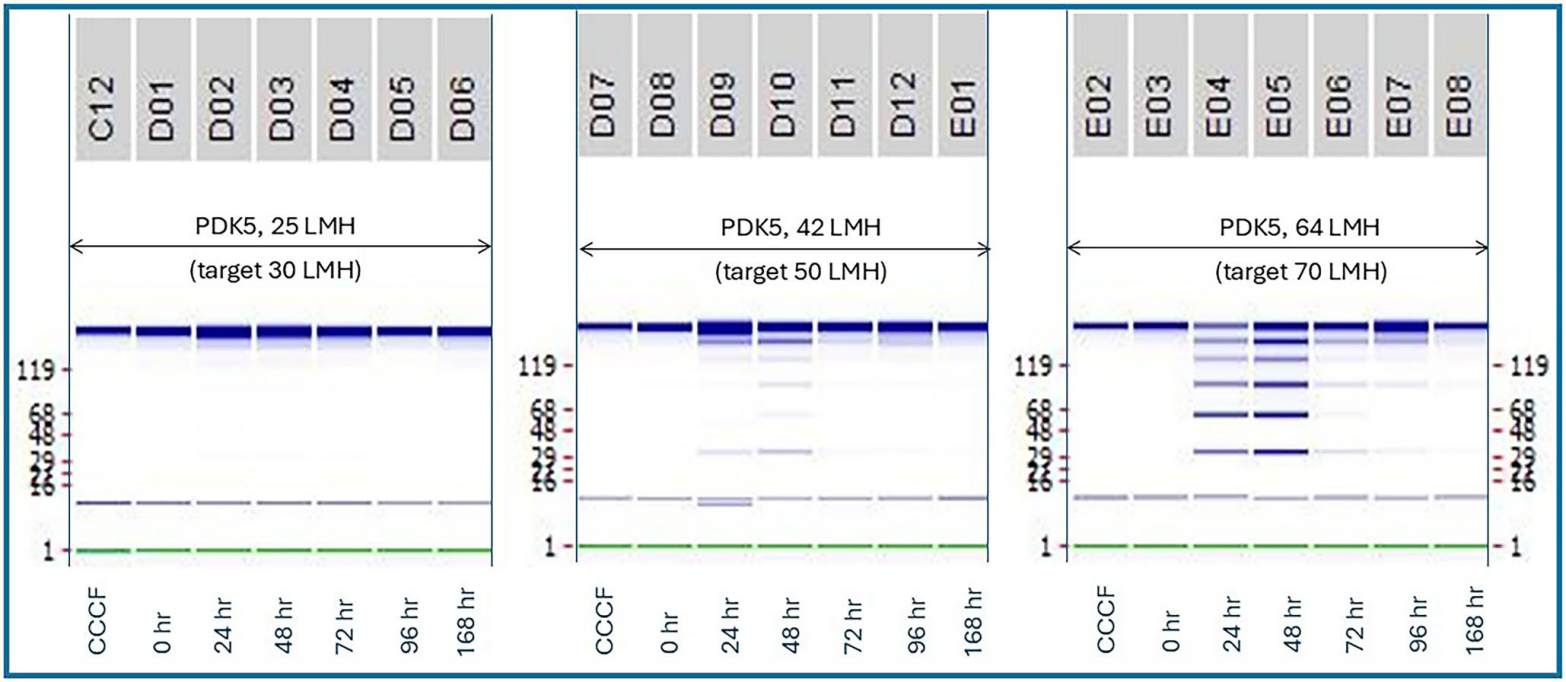
Intactness of antibodies clarified at different filtration fluxes. Time‐point samples of mAb “D” culture clarified at 25, 50, and 70 LMH on a 22‐cm^2^ Cytiva PDK5 filter and kept in an oxygen‐depleted environment before further purification on Protein A affinity resin and separated on a CE.

Timepoint samples were collected from oxygen‐depleted CCCF and supplemented with 25 mM of N‐ethyl‐maleimide (NEM) (Sigma, St Louis, MO, USA) before freezing and storing at −70°C until CE‐SDS analysis. Time‐point samples from “mAb D” CCCF treated as described above were analyzed, and images of electrophoretic profiles are shown in Figure 8. For this antibody cell culture, clarifying it at a low‐end target flux (left panel) did not lead to any noticeable DSBR of the antibody products. In contrast, clarifying it at the mid‐ and high‐limit target flux (middle and right panels, respectively) led to a noticeable DSBR. In the case of the 50 LMH target flux panel, reductions of antibody chains could be observed in the 24‐ and 48‐h point time samples and then appeared to reform starting at the 72 h time‐point. Similarly, in the high‐flux panel, the DSBRs started at 24 h and extended to the 48 h time‐point in greater proportion before starting to reform. The reduced HC and LC did not appear to be completely reformed by the 96‐h time‐point.

O'Mara et al. reported that depth filtration can induce DSBR through a ΔP‐associated cell‐lysis mechanism and evaluated primary 3 M™ Zeta Plus™ filters (10SP02A or 30SP02A) in series with 90ZB05A as the secondary filter. In our study, we likewise observed that more restrictive depth‐filtration conditions were associated with DSBR, and we use Figure 6 to provide side‐by‐side examples of restrictive versus less restrictive depth‐filter trains and the corresponding DSBR outcomes. We observed similar DSBR with the “mAb D” clarified cell culture. The DSBR patterns generated by the 3 M™ Zeta Plus™ Depth Filter 10SP02A + 60ZB05A combination at 100 LMH target flux (Figure 6, middle panel) were similar to those generated by Cytiva PDK5 filter at mid‐ and high‐fluxes (see Figure 8, center and right panels). In contrast, the same cell culture clarified on 3 M™ Harvest RC (Figure 6, right panel) to a terminal differential pressure of 5 psi and processed by this reduction assay did not show any discernible pattern of DSBR.

#### 2.3.5 Model prediction across filter types

The predictive capability of the bench‐scale process model arises from reproducing conditions when DSBR is observed with large‐scale clarification, not associated with any specific filter brand. By applying elevated differential pressure, low DO and a hold time at bench scale, the model reproduces key conditions of large‐scale clarification, including cell shear, cell lysis, reductase release, and oxygen depletion. These conditions collectively promote NADPH‐mediated antibody reduction observed during scaled up harvest.^12^, ^13^, ^14^ Consistent with findings reported in literature,^10^ smaller nominal pore range depth filters (e.g., 10SP02) generate more cell shear and cell lysis than large pore depth filters (e.g., 05SP01), and this increases the DSBR likelihood in the CCCF. The filter size area in the bench‐scale model is not a determinant factor of its predictive power. What matters is generating an equivalent differential pressure, cell cake formation, cell shear and cell lysis. We did not systematically vary cell viability in our study.

### 2.4 Clarification endpoint as a disulfide bond reduction mitigation tool

**FIGURE 9:**
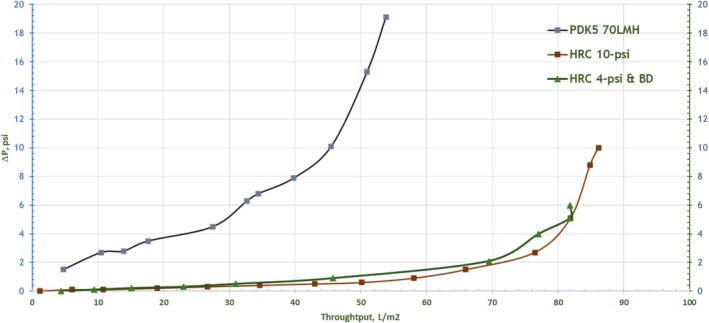
Differential pressure (ΔP) versus volumetric throughput of antibody “E” during clarification using PDK5 (control, 70 LMH flux) and Harvest RC (100 LMH flux). Five Harvest RC clarifications were performed (to a terminal ΔP of 4, 6, 8, 10, and 4 psi then blow‐down to 6 psi). For visual clarity, representative Harvest RC ΔP versus throughput profiles are shown here for two conditions (10 and 4 psi then blow‐down to 6 psi ΔP). The pooled sample at the end of each clarification was then processed as described in Figure 10.

**FIGURE 10:**
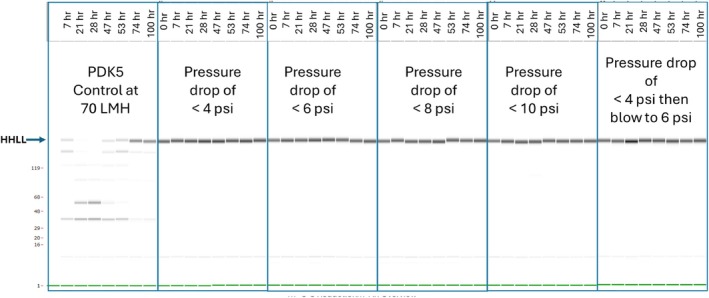
Aliquots of mAb “E” cell culture were clarified by Cytiva PDK5 (left panel), or by 3 M™ Harvest RC Chromatographic Clarifier to a pre‐defined terminal ΔP of 4–10 psi (2nd – 5th panels) without any blow‐down. In the last panel, the cell culture was clarified to 4 psi ΔP then blown down to 6 psi ΔP. Following clarification, CCCFs were kept in an oxygen‐depleted environment. Time‐point samples were collected at 0, 7, 21, 28, 47, 53, 74, and 100 h, then processed for the DSBR assay.

To better delineate the DSBR potential of the antibody product recovered for a cell culture clarified with 3 M™ Harvest RC Chromatographic Clarifier, we clarified a series of cell culture aliquots to a pre‐defined terminal differential pressure of 4, 6, 8 and 10 psi without any blow‐down. In addition, an aliquot was clarified to 4 psi ΔP then blown down to 6 psi ΔP and pooled. A control was carried out with Cytiva PDK5 depth filter at a flux of 70 LMH. Because the ΔP versus throughput behavior was similar across the five Harvest RC setpoints, Figure 9 shows representative ΔP versus throughput profiles for two of the Harvest RC cases (to terminal 10 psi ΔP, and 4 psi followed by blow‐down to 6 psi ΔP) alongside the PDK5 control. The CCCF sample used for the DSBR time‐course (Figure 10) was collected as pooled filtrate after each clarification endpoint was reached (terminal ΔP setpoints). The CCCF was subjected to low‐oxygen hold, and timepoint samples were collected for DSBR analysis.

The time‐point samples from various CCCF aliquots were processed for their DSBR potential and the results are shown in Figure 10. The antibodies in the cell culture were reduced when clarified by the control PDK5 filtration, but not in any of the cell culture clarified by 3 M™ Harvest RC to a terminal ΔP of 10 psi. To mimic at‐scale specification of clarifying to 3 psi ΔP then blowing it down to 5 psi ΔP, we clarified an aliquot of the culture to 4 psi ΔP then blew it to 6 psi ΔP. As shown in the last panel, we did not observe any DSBR of the antibody clarified under this condition.

### 2.5 Disulfide bond reduction in single‐stage AEX fiber–clarified cell culture

**FIGURE 11:**
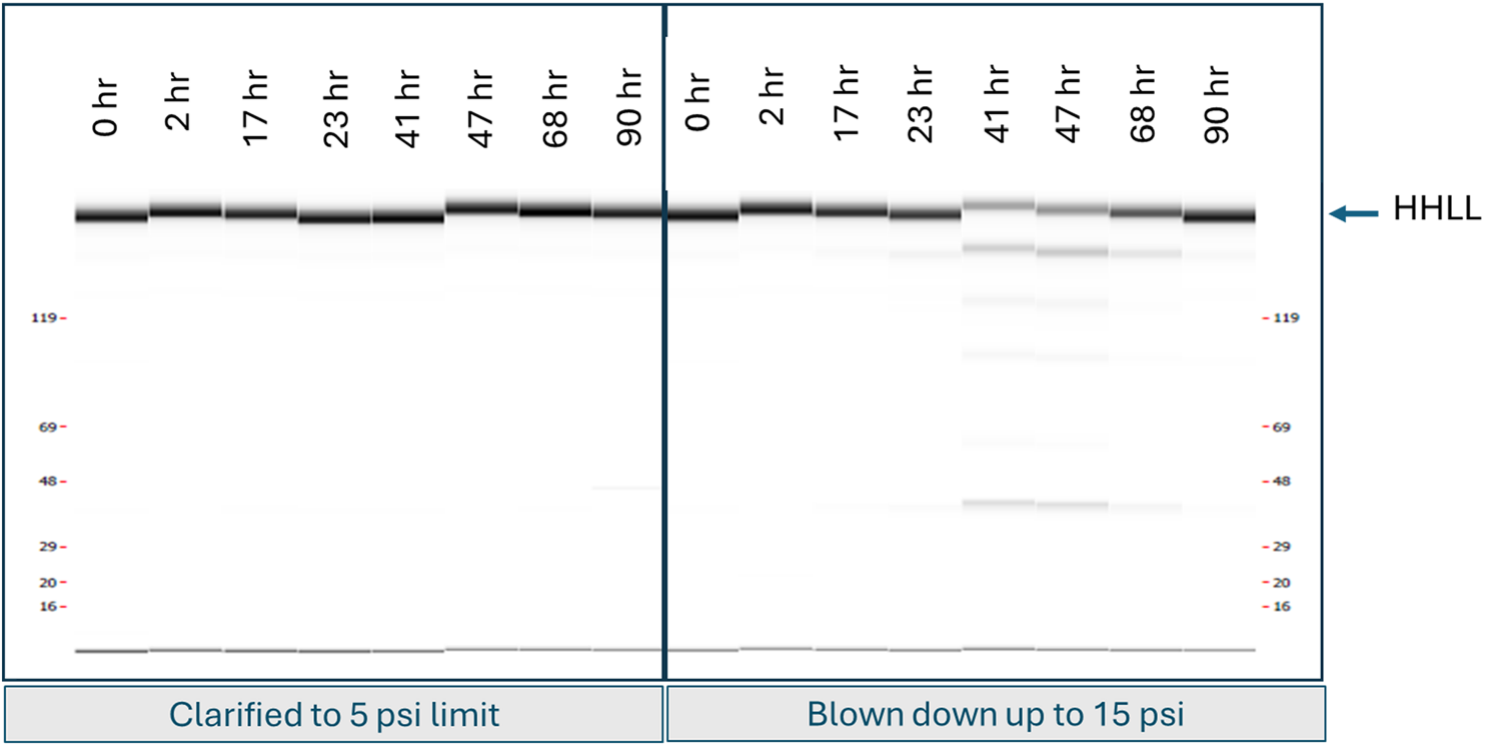
Cell culture fluid clarified by 3 M™ Harvest RC Chromatographic Clarifier to 5 psi (left panel) and the material recovered as a separate fraction following blowdown to 15 psi (right panel) were subjected to reduction assay as described in Methods. Samples were held in an oxygen‐depleted environment; time‐point samples were collected at 0, 2, 17, 23, 41, 47, 68, and 90 h then processed for the DSBR assay.

To further characterize the differential pressure of 3 M™ Harvest RC Chromatographic Clarifier, we took an aliquot of the culture and clarified it using the fiber matrix to a terminal ΔP of 5 psi. The cell culture remaining in the filtration path was then blown down to a 15‐psi terminal ΔP and collected in a separate container. The two CCCF treatment samples were then subjected to DSBR assay. The results are shown in Figure 11. The antibody filtrates collected at < 5 psi ΔP setpoint did not show any detectable reduction. However, the filtrate blown down to 15 psi ΔP was reduced when subjected to a low‐oxygen environment for a duration of 4 days.

**FIGURE 12:**
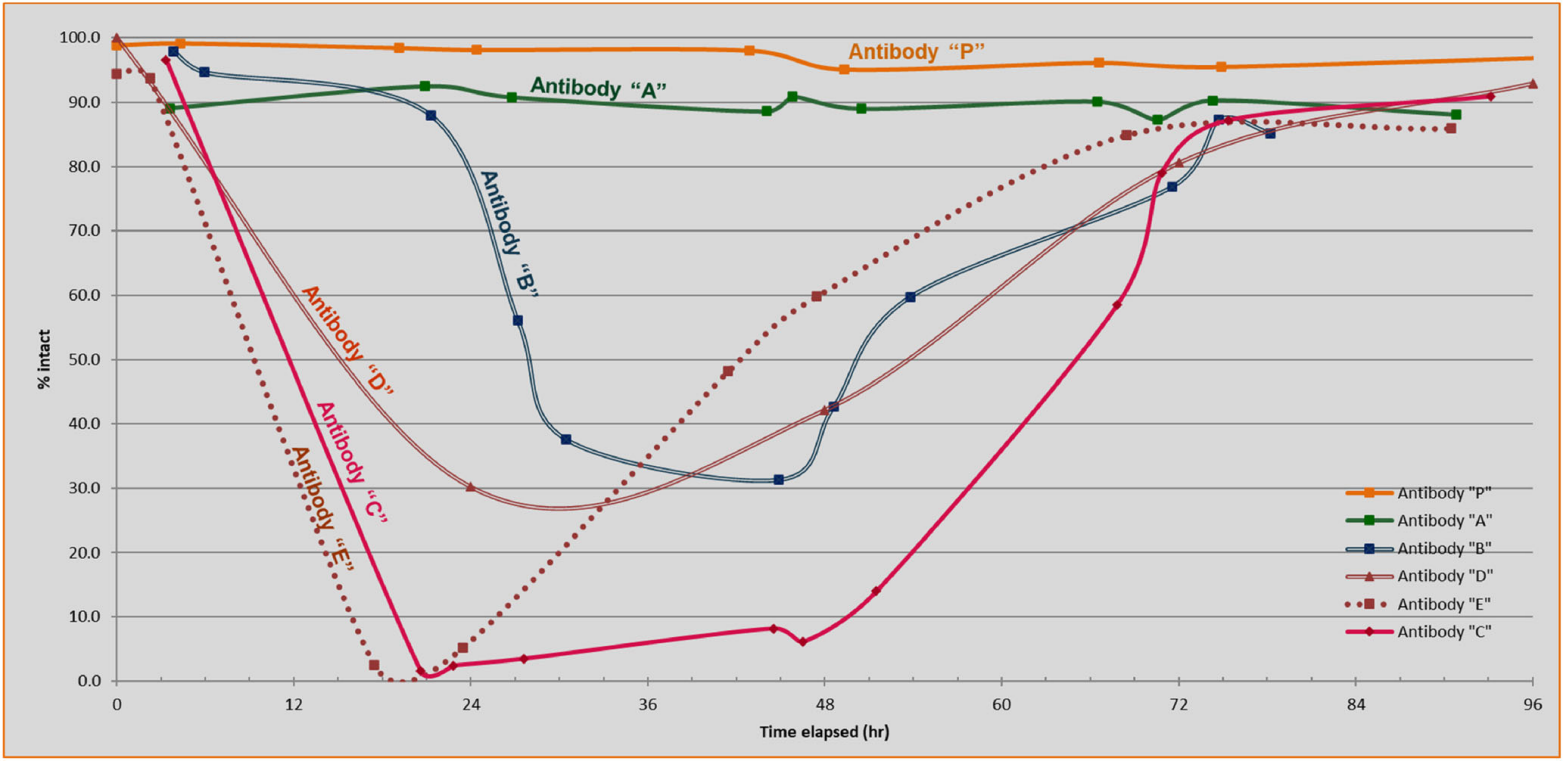
The percentage of intact antibody affinity purified from CCCF that has been subjected to DSBR assay as a function of the number of hours stored in an oxygen‐depleted environment.

By applying this DSBR assay to a panel of antibody cell cultures and quantifying the amount of intact antibody following their storage in an oxygen‐depleted environment, we were able to estimate the relative likelihood or potential of each of these cell cultures to undergo DSBR. As a reference, both “mAb B” and “mAb D” have been shown to undergo DSBR at 2000 and 2500 L scale, respectively. As such, any cell culture that retained a lower proportion of intact antibody within the 12–48 h of oxygen‐depleted exposure is considered to have a high risk of at‐scale disulfide reduction. For example, both “mAb C” and “mAb E” cell cultures were likely to incur the risk of DSBR if no mitigation measures were taken (Figure 12). Conversely, the 2kL clarification of antibody “A” was previously implemented with known DSBR mitigating measures before the availability of this bench‐scale process model assay. However, once antibody “A” was shown by this assay to exhibit lower DSBR potentials, the mitigating measures were removed from at‐scale clarifications, thus simplifying the process. No subsequent DSBR of clarified antibody “A” at manufacturing scale clarification was observed.

### 2.6 Conclusions

We evaluated 3 M™ Harvest RC Chromatographic Clarifier, a synthetic AEX fiber clarification system, on CHO cell culture at various cell densities expressing IgG_1_, IgG_2_, and IgG_4_ mAbs. The new filter utilized positively charged ligands to capture cells, cell fragments, and soluble cell culture impurities. In side‐by‐side comparisons of filtrates from more than eight different antibody cell cultures with packed cell volumes ranging from 4% to 18%, we found that the one‐stage fibrous anion exchange clarification performance met or exceeded the performance of the conventional two‐stage depth filtration systems in terms of product recovery, removal of process‐related impurities, and volumetric throughput (7, data not shown). The anionic charge filter was effective in removing > 90% of Host Cell DNA. Although the removal of Host Cell Proteins was more variable, ranging from negligible to 25%, the anionic charge was relatively more effective in removing HCPs than 3 M™ Zeta Plus™ Depth Filters when compared side‐by‐side.

The product quality attributes of antibodies clarified by either depth or AEX fiber matrix were comparable. We found the monomer content, charged variants, purity, and oligosaccharide maps of Protein A eluates were comparable. These antibody attributes remained comparable when processed to the drug substance stage.

We also found that the lower operating differential pressure of the anion exchange fiber clarification approach provided us with a mitigating tool to process antibody cultures likely to undergo disulfide bond reduction. In a 200 L clarification comparability study, we observed the antibodies purified with 3 M™ Harvest RC were not reduced whereas those clarified with 3 M Zeta Plus™ Depth Filters at the same time were reduced.

We developed a bench‐scale DSBR model for predicting disulfide bond reduction to better understand the phenomenon as early as possible during process development. It has been observed by several workers that the DSBR would typically be observed only at large‐scale production, by which time it is too late to add processing measures to prevent DSBR^10^, ^12^, ^13^ without impacting project timelines. Consequently, without any information about the DSBR risk of an antibody, manufacturing processes incorporate multiple mitigation measures at both harvest and clarified cell culture fluid stages before affinity purification of the materials.^12^ These mitigation measures without confirmation of any DSBR at manufacturing scale add operational complexity, so having a bench‐scale DSBR assay can guide process developers to design an appropriate clarification scheme.

Our results indicate that DSBR risk is strongly associated with achieving DSBR‐driving clarification conditions (elevated ΔP/cake compaction and low‐oxygen hold), and that filter pore‐range/architecture influences whether those conditions are reached. The bench assay therefore provides a practical way to evaluate DSBR risk and mitigation strategies across clarification trains by mapping outcomes to ΔP versus throughput behavior rather than filter brand alone.

We observed antibody DSBR at manufacturing scale in one case, and consequently a bench‐scale assay was developed to model the phenomenon using bench‐scale depth filters with material from 2 to 2000 L harvest cell culture. We showed that the DSBR phenomenon could be replicated with selected bench‐scale depth filters, which agrees with the observations by other workers.^10^ DSBR was observed when culture was clarified by 3 M™ Zeta Plus™ 10SP02 filter (that has a pore size range of 7–1 μm) but not when clarified by 3 M™ Zeta Plus™ 05SP01 (that has a pore size range of 10–1.5 μm). There were more cells lysed in the 3 M™ Zeta Plus™ 10SP02 filtrate (as assayed by LDH) than in the 3M™ Zeta Plus™ 05SP01 filtrate (vendor's note and data not shown). There are more cell breakthroughs of intact cells in the 05SP01 filtrate (as assayed by turbidities). A second component of the Cytiva PDK5 filter was in the range of 3.5–1.5 μm pore size. Thus, we reasoned that CHO cells with a diameter of > 10 μm were most likely to be lysed in this filter.

We used Cytiva PDK5 filters in our DSBR assay as it was easier to set up. The assay was then used to evaluate mitigation measures (data not shown) so that they could be implemented effectively and consistently during process development and clinical productions.

Using a bench‐scale disulfide bond reduction assay, we showed an antibody was reduced when clarified with the two‐stage 3 M™ Zeta Plus™ depth filter system, but not with the anion clarification system. We were able to rank the relative disulfide bond reduction potentials of various antibodies using the bench‐scale model assay and showed the reduction‐mitigating impacts of the fibrous filter on a reduction‐prone antibody at 2000 L processing scale. We observed that the charge filter could be operated to < 10 psi differential pressure without any DSBR. However, when the clarification flow path was blown down to 15 psi differential pressure, DSBR was observed. This observation suggests that one approach for mitigating DSBR at‐scale is to control terminal differential pressure without sacrificing volumetric throughput. The newer anionic fiber clarification system offers such a tool. Additionally, it is plausible that the AEX fibrous filter could capture and deplete reductases^12^, ^13^, ^14^ that cause DSBR as they have acidic isoelectric points. Based on these sets of observations and assumptions, we implemented clarification of two cell cultures that exhibited a high probability of DSBR with the anionic charge filters and observed no DSBR at 2000 L clinical production scale.

Process intensification is an important measure to advance the biopharmaceutical process development and manufacturing to the next stage of performance. However, as the performance of cell cultures is pushed to a higher level, additional factors must be considered during the harvest and purification of a biopharma product candidate. As we have expounded in this study, cell shear and its effect on product quality is a key process aspect that needs to be considered when building a modern bioprocess platform. We found that reproducing the harvest filtration conditions at bench scale allowed us to predict the DSBR likelihood after scale up and preserve active product. Novel clarification techniques that adhere to downstream process development principles can help to minimize cell lysis and shearing effects that impact product quality across different cell cultures and product candidates.

## 3 MATERIALS AND METHODS

### 3.1 Cell culture

The recombinant antibody protein was produced in Chinese Hamster Ovary (CHO) cell fed‐batch culture using stirred tank bioreactors at different scales. Various nutrients (media and feeds) and a set of process parameters such as dissolved oxygen (DO), pH, and temperature are controlled and monitored to ensure optimal cell growth and protein production in the bioreactor culture. The protein of interest is secreted into the cell culture where it accumulates; therefore, to harvest the protein of interest is to remove the cells typically by depth filtration or centrifugation.

### 3.2 Clarification

The recombinant protein was recovered from the HCCF using 3 M™ Zeta Plus™ depth filtration or chromatographic clarification in series with 0.2‐micron membrane filtration (Sartorius). The clarification filters were either with 3 M™ Zeta Plus™ depth filter Capsules 05SP01A and 60ZB05A combination or 3 M™ Harvest RC Chromatographic Clarifier. The membrane filter was a 0.2‐micron PES Sartopore 2 filter (Sartorius). The clarification devices were flushed with purified water at 60 L/m^2^ for depth filter and 20 L/m^2^ for the Harvest RC charge filter before use.

The clarification flow rates were controlled using peristaltic (Watson‐Marlow) at 2 to 200 L scale and with diaphragm pumps (Quattroflow) at 2000 L‐scale processing, respectively. The CCF clarification flux for the 05SP01A depth filter and for the 3 M™ Harvest RC Chromatographic Clarifier charge filter was 100 LMH. The flux for 60ZB05A was 200 LMH. The in‐line pressures were measured and used to calculate the differential pressures across each filter. For depth filtration, the differential pressure was kept under 8 psi. For 3 M™ Harvest RC Chromatographic Clarifier charge filtration, the terminal ΔP was 5 psi for the 25‐cm^2^ scale‐down capsule, and 3 psi for the large 1.6 m^2^ train. After product filtration, the devices were blown down dry to displace the hold‐up volume in the system at half the starting flux that is, 50 LMH. The terminal blow‐down ΔP was 10 and 5 psi, respectively, for the 25‐cm^2^ capsules and 1.6 m^2^ filtration train.

### 3.3 Bench‐scale DSBR assay

Cell culture fluid of CHO cells expressing a monoclonal antibody of interest at 16–20 million cells per mL and viabilities at 80%–90% were filtered through 22‐cm^2^ Pall PDK5 at target flux of 25, 50 and 70 LMH. When 3 M™ Zeta Plus™ depth filter 05SP01A, 10SP02A and 60ZB05A filters were used, they were clarified at 100 LMH (3 M, USA). Terminal differential pressures across Harvest RC charge filters were as described in figure legends and text. CCCF was transferred to 500‐mL Nalgene bottles fitted with Tri‐port bottle top. The oxygen was displaced with nitrogen gas; measurements were taken with BGA Analyzer until the oxygen level was 40 mmHg or below at 37°C. Samples were taken at least once a day for at least 72 h. Replicate timepoint samples were frozen in the absence or presence of 25 mM NEM (N‐ethyl‐maleimide) at −70°C until processed for analysis. Complete set of timepoint samples were thawed and affinity‐purified on 50 μL PreDictor MabSelectSure LX plate (Cytiva, USA). Following purification, the samples were diluted to 2 mg/mL and mixed 1:1 with a 2 mM NEM stock and incubated for 30 minutes at ambient temperature. The samples were then loaded on a Caliper plate and processed on LabChip GXII for further analysis (PerkinElmer, USA).

## 4 ANALYTICAL MEASUREMENTS

### 4.1 Analytical protein a chromatography

Analytical Protein A chromatography was used to determine the concentration of IgG protein in harvest before and after clarification. A Poros Protein A column (Thermofisher, USA) was used on an Agilent HPLC 1200 series (Agilent, USA). The mobile phase A was 50 mM sodium phosphate, 150 mM NaCl, pH 7.0. The mobile phase B was 25 mM sodium phosphate, 150 mM NaCl, pH 2.5.

### 4.2 Imaged capillary isoelectric focusing (iCIEF)

The iCIEF technique analyzes charge heterogeneity in protein preparation. Changes in charge may reflect deamidation, aggregation, isomerization, fragmentation, glycation of lysine residues, and/or glycan sialyation, and so forth. This assay was executed with iCE3 System (Bio‐techne, USA) and Alcott 720NV autosampler unit (ProteinSimple, USA).

### 4.3 Capillary gel electrophoresis (cGE) analysis

Capillary Gel Electrophoresis (cGE) is an analytical separation method where charged molecules are separated in capillaries filled with a porous gel matrix. cGE is used to separate large biological molecules like proteins, DNA, and RNA. The cGE assay was executed with AB Sciex Beckman PA800 Plus Pharmaceutical Analysis capillary electrophoresis (CE) system.

### 4.4 Oligosaccharide mapping

The glycosylation patterns of monoclonal antibody drugs are assessed by a combination of enzymatic cleavage to release the N‐link oligosaccharides from the antibody followed by detection of these sugars by liquid chromatography. The cleavage and subsequent fluorescent labeling of N‐link oligosaccharides is accomplished using the enzyme PNGase and dyes from the GlykoPrep kit (Prozyme, USA). The labeled glycans are then analyzed by HPLC using a hydrophilic interaction liquid chromatography column (HILIC) to separate the fluorescently labeled glycans.

### 4.5 Host cell protein (HCP) ELISA analysis

The HCP levels were quantified using Cygnus CHO HCP 3rd Generation F550 (Cygnus Technologies, USA). The standard curve was constructed using a four‐parameter logistic fit.

### 4.6 Protein concentration by slope analyses

SoloVPE Agilent Technologies Cary 60 UV–Vis was used for spectrophotometry analysis. Small UV Disposable Vessels (Brand C Technologies, INC Cat. # OC0009‐1‐P50) were used for sample UV absorption reading.

### 4.7 Protein concentration by DropSense 96 analyses

DropSense 96 was used for spectrophotometry analysis. DropPlate 96‐D+ was used for sample UV absorption reading.

### 4.8 Dynamic light scattering (DLS)

DLS measures the size of a particle. It detects the changes in the intensity of light scattered by a particle. Both UNCLE from Unchained Labs (Pleasanton, CA) and MicroTrac (San Diego, CA) were used for this assay.

Turbidity of feed and filtrates was measured using Thermo Scientific Orion AQ4500. To qualitatively assess the DNA levels of bioprocess fluids, 1 M acetic acid was added to filtrate solutions as described previously.^4^ The ratio of acidified turbidity to non‐acidified turbidity correlates to DNA levels in the fluid.

## AUTHOR CONTRIBUTIONS

**Hendri Tjandra:** Conceptualization, methodology, investigation, project administration, resources, writing – original draft, writing – review and editing, visualization, data curation, supervision, validation, formal analysis, software. **Deepa Surendar:** Investigation, validation, formal analysis, software, methodology. **Isu Yoon, Florence Rusly, Yan Su:** Investigation. **Vu Thai:** Investigation, formal analysis, methodology. **Yi Liu:** Investigation, conceptualization, formal analysis, supervision, writing – review and editing, resources, methodology, project administration. **Ashley Hesslein:** Funding acquisition, project administration, resources.

## CONFLICT OF INTEREST STATEMENT

All of the authors were employees of Bayer Healthcare LLC during the time the work was performed. The authors are not aware of any affiliations, memberships, funding, or financial holdings that might be perceived as affecting the objectivity of this work.

## Data Availability

The data that support the findings of this study are available from the corresponding author upon reasonable request.
